# The relationship between cancer information burden, attitudes, and behaviors toward cancer screenings in women aged 30–70 years

**DOI:** 10.1590/1806-9282.20241395

**Published:** 2025-03-31

**Authors:** Tuğba Solmaz, Aygül Kıssal

**Affiliations:** 1 Tokat Gaziosmanpaşa University, Faculty of Health Science, Department of Nursing – Tokat, Türkiye.

**Keywords:** Early diagnosis of cancer, Breast cancer, Cancer of cervix, Colon cancer, Attitude

## Abstract

**OBJECTIVE:**

The aim of this study was to evaluate the relationship between cancer information burden, attitudes, and behaviors toward cancer screening in women aged 30–70 years who applied to family health centers.

**METHODS:**

The data were collected with Personal Information Form, Attitude Scale for Cancer Screening, and Cancer Information Overload Scale from 398 women.

**RESULTS:**

The mean score of the participants was 16.22±4.66 on the Cancer Information Overload Scale and 93.05±13.80 on the Attitude Scale for Cancer Screening. A statistically significant difference was found between the mean scores of the women according to their educational level, occupational status, knowledge about cancer screening, and the presence of cancer in themselves or their families (p<0.05). In addition, a strong positive but statistically insignificant correlation (r=0.061, p=0.223) was found between the Cancer Information Overload Scale and the scores of the Attitude Scale for Cancer Screening.

**CONCLUSION:**

Women had low rates of knowing the timing of colon, cervical, and breast cancer screenings, having been screened, and thinking of participating in screenings in the following year. In order to improve women's attitudes toward early diagnosis of cancer in a positive way, informative public education activities should be continued.

## INTRODUCTION

Cancers are among the most important health problems today due to their high mortality and morbidity, cost, duration, and side effects of treatment^1^. Early detection and prevention are more important than treating cancer^2^. It has been stated that most of the deaths that may occur with planned strategies for cancer can be prevented. In this context, one of the effective and cost-efficient interventions is systematic community-based screening programs. Involving the community in screening programs will be possible through intensive awareness-raising activities and informed follow-up^3^. Although early screening programs in cancer are important, many factors such as poverty, lack of access to screening services, cost, low level of education, lack of knowledge, beliefs, and attitudes toward cancer negatively affect behaviors toward cancer screening^4^. While the widespread availability of information on cancer facilitates access to accurate and reliable information, on the other hand, information overload causes confusion. Cancer information burden, a concept that has emerged in recent years, defines the intensity and burden of information that individuals have about the disease. It has been suggested that with the increase in the burden of cancer information, there may be a decrease in health protective and cancer preventive behaviors of individuals, a decrease in participation in screening, and difficulty in compliance with treatment^5,6^. Knowledge about this issue is limited. In a study conducted in the USA, it was found that as the burden of cancer knowledge increased, individuals exhibited negative behaviors toward cancer and behaved fatalistically^7^. In another study, it was stated that the increase in cancer information burden caused uncertainty in individuals, and as a result of this situation, they avoided accessing accurate information about cancer and moved away from preventive health behaviors^8^. It is important to determine the factors affecting the participation of individuals in cancer screening and to eliminate the factors that prevent screening^9^. In order to reduce the burden of cancer on society, health professionals should evaluate the knowledge, attitudes, and behaviors of the community they care for regarding cancer screening^6^. There are a limited number of studies in the literature on the relationship between women's cancer information burden and their attitudes toward screening. In line with this information, the current research was conducted to evaluate the relationship between cancer information burden, attitudes, and behaviors toward cancer screening among women aged 30–70 years who applied to family health centers in a province.

## METHODS

### Design and location of the study

The present research is a descriptive design. It was conducted in Türkiye using a face-to-face interview technique by the researcher with individuals aged 30–70 years. The sample size of the study was found to be 318, using the sample size for a known population (95% confidence interval, alpha=0.05, p=0.50, n=150.419). Considering that there may be losses during data collection, it was decided to include approximately 398 people in the study, 20% more than the sample number found in the calculation. A total of 10 family health centers in the province where the research was conducted were accepted as a cluster. Three family health centers were selected from among the available centers, which have a high frequency of visiting the physician and serve the community at low, middle, and high socioeconomic levels. The study was conducted between February 20, 2023, and June 30, 2023, with the women living in a city in northern Türkiye.

### Data collection

Data were collected using a Personal Information Form (PIF), Cancer Information Overload Scale (CIOS), and Attitude Scale for Cancer Screenings (ASCS).

### Measurement tools

PIF consists of 13 questions about the descriptive characteristics of the individuals and the status of cancer screening. The CIOS was developed by Jensen et al., and its validity and reliability were adapted by İnci et al. It includes eight items, measured on a 4-point Likert scale^6,10^. In this study, the Cronbach's alpha coefficient of the scale was found to be 0.89. ASCS was developed by Yıldırım Öztürk et al. in 2020. Turkish validity and reliability study was conducted. It includes 24 items, measured on a 5-point Likert scale^11^. In this study, the Cronbach's alpha coefficient of the scale was found to be 0.86.

### Data analysis

IBM SPSS Statistics 23.0 was used for data analysis, and the significance level was set as α=0.05. The number, percentage, score level, and standard deviation were given in descriptive analyses. The relationship between women's cancer information burden scores and their attitudes toward cancer screening was evaluated by the Pearson correlation analysis. In the analysis of independent variables according to dependent variables, Student's t-test, Mann-Whitney U test, one-way variance (=ANOVA) analysis (further analysis Tukey HSD), and Kruskal-Wallis test were used by taking normality analysis into consideration.

Ethical considerations: Institutional approval was obtained from the noninterventional clinical research ethics committee of a university (16.02.2023 / Decision Number: 23-KAEK-030) and the Provincial Health Directorate (07.04.2023 / Decision Number: 04/02). The study was conducted according to the code of Helsinki. Written and verbal consent was obtained from the participants.

## RESULTS

The mean age of the participants included in the study was 46.56±10.90 years, of which 38.4% were primary education graduates, 82.2% were married, 70.1% were housewives, and 86.7% lived with their husbands and children. The rate of those who reported having a chronic disease was 35.2%, and 37.2% of the participants did not receive information about cancer screening. The first three information sources of those who received information about cancer screening were family physicians, nurses and other healthcare workers, and specialized physicians. It was found that more than half of the participants did not know the screening times, did not participate in screening, and did not plan to participate in screening the following year (Table 1).

**Table 1:** Sociodemographic and screening-related descriptive characteristics of participants (n=398).

Variables	Categories	n	%
Age $\bar{\text{X}}\pm \text{SD}$ (46.56±10.90)	30–39	123	30.9
	40–55	179	45.0
	56–70	96	24.1
Education level	Literate	26	6.5
	Primary school	153	38.4
	High school	117	29.4
	Bachelor's degree	102	25.7
Marital status	Married	327	82.2
	Single	71	17.8
Occupational status	Housewife	279	70.1
	Worker	39	9.8
	Healthcare worker	33	8.3
	Teacher	30	7.5
	Officer	17	4.3
Living together	With spouse or children	345	86.7
	Alone	28	7.0
	Others (mother and father, sister, husband and family of husband's, friend)	25	6.3
Income level	Low	120	30.2
	Moderate	200	50.3
	High	78	19.5
Presence of chronic disease	Yes	140	35.2
	No	258	64.8
Receiving information about cancer screenings	Yes	250	62.8
	No	148	37.2
*Source of information about cancer screenings	Family physician	147	36.9
	Nurse and other healthcare worker	91	22.9
	Specialized physician	76	19.1
	Social media (televison or internet)	49	12.3
	Acquaintance/relatives	47	11.8
	Others	21	5.3
Knowing the screening time for the fecal occult blood test	I don't know	266	66.8
The fecal occult blood test screening	I didn't have it done	321	80.7
Knowing the time of colonoscopy	I don't know	256	64.3
The status of colonoscopy screening experience	I didn't have it done	338	84.9
The thought of having a colonoscopy next year	No	278	67.8
Knowing the time of mammography	I don't know	193	48.5
The status of mammography screening experience	I didn't have it done	258	64.8
The thought of having a mammography next year	No	214	57.4
Knowing the timing of the pap smear test	I don't know	199	50.0
The status of pap smear test screening experience	I didn't have it done	219	55.0
The thought of having a pap smear test next year	No	208	52.3
**Total**		398	100.0

* It was evaluated based on the percentage of responses.

SD: standard deviation.

The presence of colon, breast, and cervical cancer in first-degree relatives was found to be 3.5, 1.5, and 0.8%, respectively. The presence of colon, breast, and cervical cancer in second-degree relatives was 3.5, 1.5, and 0.8%, respectively. The rates of colon, breast, and cervical cancer in the individuals themselves are 0.5, 0.8, and 0.8%, respectively. The top three reasons for not having a cancer screening test were feeling healthy, not considering oneself at risk, and negligence (Table 2).

**Table 2:** Distribution of familial and personal presence of cancer and reasons for not being tested in participants (n=398).

Variables	Categories	Colon cancer		Breast cancer		Cervical cancer	
		n	%	n	%	n	%
Presence of cancer in the first-degree relative (mother, father, and children)	Yes	14	3.5	6	1.5	3	0.8
Presence of cancer in the second-degree relative (sister or brother, grandchild, grandfather, and grandmother)	Yes	14	3.5	6	1.5	3	0.8
Presence of cancer in oneself	Yes	2	0.5	3	0.8	3	0.8
*Reasons for not wanting to have a cancer screening test	Feeling healthy	98	24.6	83	20.9	80	20.1
	Not considering oneself at risk	73	18.3	62	15.6	56	14.1
	Negligence	49	12.3	45	11.3	37	9.3
	Absence of time	28	7.0	22	5.5	23	5.8
	Lack of information	26	6.5	18	4.5	26	6.5
	Fear of pain/pain in the procedure	20	5.0	10	2.5	13	3.3
	Embarrassment	13	3.3	12	3.0	15	3.8
	Fear of cancer diagnosis	12	3.0	7	1.8	7	1.8

* More than one option was selected. Selected options are expressed as numbers and percentages.

The CIOS mean scores of the participants according to age, educational level, occupational status, receiving information about cancer, and the presence of cancer in themselves or in their family showed a similar distribution (p>0.05). There was a statistically significant difference between the CIOS mean scores of the participants according to the income level (p<0.05). ASCS mean scores of the participants according to the age and income level were similar (p>0.05). There is a statistically significant difference (p<0.05) between the ASCS mean scores of the participants according to their education, occupational status, knowledge about cancer screening, and the presence of cancer in themselves or their families (Table 3).

**Table 3:** Comparison of the mean scores of the CIOS and ACSC according to selected sociodemographic and breast cancer characteristics of participants.

Variables		Scales	
		CIOS	ASCS
		$\bar{\text{X}}\pm \text{SD}$	$\bar{\text{X}}\pm \text{SD}$
Participants		16.22±4.66	93.05±13.80
Age	30–39	16.27±4,67	94.86±13.07
	40–55	16.34±4.71	92.63±13.64
	56–70	15.92±4.59	91.52±14.84
Significance		F=0.271 p=0.762	F=1.738 p=0.177
Education level	Literate	18.15±3.70	82.50±15.17^a^
	Primary school	15.76±4.93	90.35±12.49^b^
	High school	16.39±4.71	94.92±14.04^bc^
	Bachelor's degree	16.21±4.32	97.66±12.86^c^
Significance		KW=2.396 p=0.494	KW=23.774 p=0.000
Occupational status	Housewife	16.27±4.63	91.56±13.83^a^
	Worker	15.39±5.44	92.90±12.56^ab^
	Healthcare worker	15.58±4.29	100.18±13.91^b^
	Teacher	16.30±4.28	98.60±11.51^ab^
	Officer	18.41±3.45	94.29±13.99^ab^
Significance		KW=5.663 p=0.226	KW=17.060 p=0.002
Income level	Low	17.61±4.00^a^	94.88±13.90
	Moderate	16.36±4.60^b^	93.00±14.09
	High	13.72±4.80^c^	90.38±12.59
Significance		F=18.091 p=0.000	F=2.524 p=0.081
Receiving information about cancer screenings	Yes	16.13±4.64	95.34±13.09
	No	16.37±4.71	89.18±14.16
Significance		t=–0.481 p=0.631	t=4.403 p=0.000
Presence of cancer personal or family	Yes	16.62±4.59	95.72±13.34
	No	16.07±4.69	92.05±13.86
Significance		t=1.066 p=0.287	t=2.386 p=0.018

CIOS: Cancer Information Overload Scale, ASCS: Attitude Scale for Cancer Screening; KW: Kruskal-Wallis, F: analysis of variance, t: Paired sample t-test, $\bar{\text{X}}$ : average, SD: standard deviation,

a–c no significant difference between groups with the same letter.

In this study, a strong positive but statistically insignificant relationship (r=0.061, p=0.223) was found between the CIOS score and ASCS scores.

## DISCUSSION

Recent studies have argued that cancer information burden may cause fear, anxiety, and stress in individuals and may be effective in participation in cancer screening^8,12^. In this study, it was reported that more than half of the participants received information about cancer screenings. Similarly, in the literature, the rate of more than half of the participants having information about cancer and cancer screenings was found to be high^9,13^. This information shows that the community has access to information about cancer. However, in this study, health professionals were found to be low-level sources of information about cancer screening. Similar results have been reported in other studies^2,14^. The relationship of trust between the health worker and the patient is established through clear, accessible, and sometimes culturally appropriate communication and information about the screening program. One study has emphasized that healthcare providers play a more active role in screening and reports that a patient who receives a doctor's recommendation is four to five times more likely to be vaccinated against HPV^15^. It can be considered that informing activities performed by healthcare professionals about cancer screening programs is an important intervention to increase the participation rate of women in screening. In this study, the most common factors reported among the reasons for participants not performing early cancer diagnosis practices were the thought of being healthy, not considering oneself risky, and negligence. Similarly, in the literature, it is reported that the reasons for not participating in cancer screening are the thought of being healthy^16^, not considering oneself risky^17^, negligence^18^, functional or physical limitations, and even lack of access to health services^19^. It has also been reported that cultural or religious beliefs and practices may influence knowledge, attitudes, and precautions related to screening and may make it difficult to comply with health seeking or resist public health planning interventions^19,20^. The findings obtained may indicate that the women participating in the study need to be better informed about cancer risks, encouraged to participate in screening programs, and need effective educational activities. For this purpose, it is recommended that healthcare professionals should increase educational activities that can positively affect attitudes toward cancer screening in women and implement initiatives that can make screening programs a part of medical examinations.

The CIOS mean score of the participants in the study was 16.22±4.66. Considering that min:8, max:32 points were obtained from the scale, it can be said that women's cancer information burden is at a moderate level. Similarly, it was found in the literature that women's cancer information burden was at a moderate level^5,6^. The moderate level of cancer information burden of participants may suggest that they were not exposed to excessive cancer burden. In this study, the attitudes toward screening tests of the participants who stated that they had knowledge about cancer screening tests were also found to be more positive. Similar to the results of this study, it has been reported in the literature that the level of knowledge positively affects the attitude toward cancer screening tests^21,22^. In another study, an illustrated leaflet and a FAQ (Frequently Asked Questions) section were used to guide the patient through the procedure for cervical cancer screening. A doctor is also available to answer any questions that patients may have. This suggests that receiving information about the procedure is a factor that facilitates women's participation in screening^23^. This can be considered as evidence of the importance of individuals having information about cancer screening.

In this study, the ASCS mean score was found to be 93.05±13.80. Considering the min=24 and max=120 scores obtained from the scale, it is observed that women's attitudes toward cancer screening are above average. In a similar study, the attitudes of individuals toward cancer screening were found to be above average^24^. In different studies, the mean ASCS score of women was found to be below average^9,11^. This difference may be due to the fact that the studies were conducted on different populations. In this study, it was found that there was a correlation between the participants’ status of having cancer in themselves or in their families and the ASCS mean scores. In another study, the rate of cancer screening was found to be higher in people with a family history of cancer compared to those without a family history of cancer^2^. According to the results of this study, the presence of cancer diagnosis in first-degree relatives, the fact that the status of receiving a cancer diagnosis is significant in cancer screening, and the attitude toward cancer screening are expected to increase.

In this study, a strong positive correlation was found, although it was statistically insignificant, between the CIOS mean scores and ASCS mean scores of the participants. Although cancer information burden is a situation that increases anxiety in individuals, it increases awareness and directs individuals to take measures to protect their health^25^. This finding may suggest that as the cancer information load increases, the anxiety experienced prioritizes issues that can positively affect women's health and develops positive behavior toward cancer screening. Contrary to the findings of the present study, some studies show a decrease in cancer screening behaviors as the cancer information load increases^8,10,12^. These differences can be explained by the fact that the information burden about cancer leads to confusion in individuals and affects the attitude toward cancer screening by affecting the sense of trust in health personnel or the system.

### Limitations and strengths of the study

The most important limitation of this study is that the sample group consists of women who applied to three family health centers and participated voluntarily. Research findings cannot be generalized to all women. The limited number of studies on the subject in the literature reveals the originality of this study. One of the strengths of the study is the evaluation of the relationship between women's cancer knowledge burden and their attitudes toward screening using correlation analysis.

## CONCLUSION

In this study, more than one-third of the participants did not receive information about cancer screening. When the timing of colon, cervix, and breast cancer screening and the status of screening were analyzed, it was found that more than half of the participants did not know the screening times and more than half of the participants did not participate in screening and did not plan to participate in screening in the following year. Cancer information burden of participants was found to be moderate, and their attitudes toward cancer screening were above average. Our study revealed that attitudes toward cancer screening increased as the burden of cancer information increased in women. It is important to determine the information needs of women about screening programs and to evaluate the factors affecting participation in cancer screening.

## ETHICAL APPROVAL

Ethical permission was obtained from Tokat Gaziosmanpaşa University Clinical Research Ethics Committee (2023/02).
